# The Effects of Mild Gestational Hyperglycemia on Exclusive Breastfeeding Cessation

**DOI:** 10.3390/nu8110742

**Published:** 2016-11-19

**Authors:** Sergio Verd, Diego de Sotto, Consuelo Fernández, Antonio Gutiérrez

**Affiliations:** 1 Department of Primary Care, Balearic Health Authority, Health Sciences Research Institute IUNICS, 10 Alexandre Rosselló Ave, 07002 Palma de Mallorca, Spain; 2 Department of Primary Care, Balearic Health Authority, 07003 Palma de Mallorca, Spain; consuelofernandez@ibsalut.caib.es; 3 Endocrinology Unit, Department of Paediatrics, Hospital Son Espases, Valldemossa Road, 79, 07010 Palma de Mallorca, Spain; diego.desotto@ssib.es; 4 Molecular Biology Unit, Division of Hematology, Son Espases University Hospital, Valldemossa Road, 79, 07010 Palma de Mallorca, Spain; antoniom.gutierrez@ssib.es; 5 Palma de Mallorca Institute of Health Research (IdISPa), Valldemossa Road, 79, 07010 Palma de Mallorca, Spain

**Keywords:** breastfeeding, gestational diabetes, neonate, glucose tolerance test, prediabetes, insulin resistance, pregnancy outcomes

## Abstract

Gestational diabetes increases the risk of a range of adverse perinatal outcomes, including breastfeeding failure, but the best cut-off point for gestational diabetes is unknown. The purpose of this study was to evaluate the association between mild gestational glucose tolerance impairment and the early cessation of exclusive breastfeeding (EBF). This is an observational study of 768 women with full term pregnancies that were screened for gestational diabetes at 24–28 weeks gestation. Subjects were divided into two groups: those with a normal 1-h glucose challenge test and those with an elevated 1-h glucose challenge test but still did not qualify for gestational diabetes. We constructed multivariable logistic regression models using data from 616 women with normal gestational glucose tolerance and 152 women with an isolated positive 1-h glucose challenge test. The risk of early exclusive breastfeeding cessation was found to increase in women with mildly impaired glucose tolerance during pregnancy (adjusted OR, 1.65; 95% CI: 1.11, 2.45). Risks of early EBF cessation were also independently associated with the amount of neonatal weight loss and admission to the neonatal ward. Instead, parity was associated with a decreased risk for shorter EBF duration. Insulin resistance—even in the absence of gestational diabetes mellitus—may be an impeding factor for EBF.

## 1. Introduction 

The role of maternal metabolic health on early lactation success is only recently gaining recognition. A wide range of entangled factors may contribute to reduced milk supply. We know that gestational diabetes predisposes women to both perinatal depression and undesired weaning [1,2,3]. Several authors have reported that glucose homeostasis during pregnancy may affect breast development [4] and could correlate with breastfeeding outcomes [5,6]. Insulin resistance may act at the lactocyte level [7]. Pregnant women at risk of metabolic syndrome are prone to delayed onset of lactogenesis stage II (DLII) [8]—a risk factor for early breastfeeding cessation [9]. In recent years, it has been shown that most of the time, variation in onset of lactogenesis stage II (LII) is predicted by 1-h post-glucose challenge (1-hOGTT) at 26 weeks of gestation [8]. This finding is in line with current data suggesting that the correlation between any degree of glucose tolerance impairment and adverse offspring outcome follows a continuous pattern, and that even mild degrees of hyperglycemia may be associated with complications [10].

Taken together, these data suggest that suboptimal maternal glucose tolerance may be a key factor in the establishment of breastfeeding. Despite the rising prevalence of gestational prediabetes, studies on neonatal outcomes of this condition are still scarce. To shed some light on this issue, we conducted a secondary analysis and evaluated the following research question: is there an association between mild gestational hyperglycemia and the early cessation of exclusive breastfeeding (EBF)? 

## 2. Experimental Section

### 2.1. Enrollment

We enrolled a population-based sample of mother–infant dyads attending a general care pediatric clinic in a middle-class neighborhood in Majorca, Spain. The enrollment phase lasted from January 2007 to December 2012. We invited all mothers who attempted breastfeeding to participate in ‘‘a study on infant feeding’’ upon their first well-child visit. The study was approved by the Institutional Review Boards of the Health Authority of the Balearic Islands, and participants consent was obtained prior to their inclusion in the study. 

### 2.2. Selection Criteria and Data Collection

This analysis was conducted in the context of an ongoing observational study of breastfeeding natural history. The study protocol has previously been described in detail [11,12]. In brief, the study was conducted as a review of medical records from a Pediatric Office where approximately 120 babies are enrolled every year. Prenatal inclusion criteria were: (1) the routinely administered 24 to 28-week gestation 1-hOGTT, and (2) mothers had to be free of gestational diabetes (GDM). Post-birth inclusion criteria were (3) delivery at term (37 weeks of gestation), and (4) the mother initiated breastfeeding as planned. We extracted additional data on patient characteristics and on infant feeding from the prenatal and pediatric medical records. From the pediatric record, information on infant feeding was collected at discharge from the hospital and at the scheduled well-baby visits. Information about the duration of EBF was obtained from pediatric records, information previously collected from mother’s report of how the baby was being fed at the time of well-baby visits at 2 weeks, and at 2, 4, 6, and 9 months of age. Duration of EBF was defined as the last notation in the record when the infant was fed only breast milk. According to the WHO, exclusive breastfeeding means that the infant receives only breast milk. No other solids are given, with the exception of vitamins, minerals, or medicines. Of the eligible mother–infant dyads, 768 had information available for all key variables, and were therefore included in the analyses presented here.

### 2.3. Glucose Challenge Test

According to the recommendations of the American College of Obstetrics and Gynecology (ACOG) [13], standard practice in our setting involves universal screening for GDM in all pregnant women at 24–28 weeks’ gestation by a nonfasting 1-h 50 g glucose challenge test. Patients testing positive for the 1-hOGTT (1-h plasma glucose 7.8 mmol/L) were asked to return for a 3-h 100 g oral glucose tolerance test (3-hOGTT).

Categorization. Based on 1-hOGTT and 3-hOGTT, subjects were stratified into the following three glucose tolerance groups:

(i) Normal glucose tolerance (NGT), defined by normal 1-hOGTT results (1-h plasma glucose < 7.8 mmol/L);
(ii) Mild impairment of glucose tolerance (MIGT), defined by a single abnormal value greater than or equal to 7.8 mmol/L, but less than 10.6 mmol/L;
(iii) GDM, requires at least two of the following on the 3-hOGTT: fasting glucose 5.8 mmol/L, 1-h glucose 10.6 mmol/L, 2-h glucose 9.2 mmol/L, or 3-h glucose 8.1 mmol/L.

### 2.4. Data Analysis

Our outcome measure was discontinuation of EBF. Dichotomization: this variable has been split at the median to form short and normal breastfeeding duration groups. Our primary predictor was MIGT in pregnancy. Patients were allocated to the NGT group or to the MIGT group. Data were analyzed using the IBM-SPSS (V22.0) Package (IBM Corp., Armonk, NY, USA). First, we examined distributions and summary measures for each variable. Table 1 shows, for each study group, continuous variables as median followed by range, while categorical variables are presented as proportions. Univariate differences across the groups were assessed using Mann–Whitney test for continuous variables, and either chi square or Fisher’s exact test for categorical variables. Multiple regression analysis was used to identify perinatal factors that independently predicted a short duration of EBF. Covariates considered included parity, glucose tolerance status in pregnancy, delivery type, birth weight, admission to the neonatal ward, and neonatal weight loss at discharge. A series of models were constructed using these covariates. The final models included main effects significant at *p* < 0.05.

## 3. Results

### 3.1. Patient Characteristics

Table 1 shows the demographic and perinatal characteristics of the 768 study participants stratified into our two predefined glucose tolerance categories in pregnancy: NGT (*n* = 616) and MIGT (*n* = 152). Around eighty percent (80.2%) of subjects tested in the normal range for glucose tolerance. This was a predominantly middle-class cohort from a general pediatric clinic where most participants had private health insurance. There were no significant differences between the groups with respect to most baseline characteristics, but birth weight was lowest in the NGT group (*p* = 0.018), and birth height was lower in the NGT group than in the MIGT group—though this difference did not reach statistical significance (*p* = 0.13).

### 3.2. EBF Outcome: Univariate Analysis

Table 2 shows that, upon univariate analysis, the rate of EBF adverse outcomes was significantly higher in the MIGT than in the NGT group (58% vs. 48%; *p* = 0.03). In fact, in the MIGT cohort, median duration of EBF was 56 days (1–300), while in the NGT cohort, it was significantly longer: 100 days (1–360) (*p* = 0.045). Other significant findings for decreased EBF duration included parity, gender of the newborn infant, neonatal weight loss at discharge, and admission to the neonatal ward.

### 3.3. EBF Outcome: Multivariate Analysis

We reanalyzed the association between MIGT and EBF outcome after adjustment for significant univariate variables (Table 3). EBF duration—as a dichotomous variable (<100 days)—was independently associated with the MIGT group (OR: 1.65; CI: 1.11–2.45). In addition, neonatal weight loss at discharge and admission to the neonatal ward were identified as significant risk factors for short EBF duration in the multivariable model. Instead, parity was associated with a decreased risk for shorter EBF duration.

## 4. Discussion

In this report, we aimed to determine whether an abnormal 1-hOGTT is independently associated with adverse EBF outcomes despite a normal subsequent 3-hOGTT. We have shown that MIGT during pregnancy predicts a shortened breastfeeding duration. To our knowledge, there is only one other study on the lactational implications of mild gestational hyperglycemia. Interestingly, it shows that testing positive in the 1-hOGTT is the main contributing factor to a delay or failure in the onset of LII [9], and specifically, that DLII is a clinical indicator of women at risk of early postpartum breastfeeding cessation. Three groups have evaluated the effect of DLII on breastfeeding outcomes. Chapman et al. showed that among women planning to breastfeed ≥6 months, women without DLII were more likely to continue breastfeeding [14]. In the analyses by Hruschka and colleagues, immediate postpartum supplementation was associated with DLII [15]. Finally, data from over 2400 mothers showed that DLII was associated with the cessation of any and exclusive breastfeeding at 4-weeks postpartum [9]. 

Since O’Sullivan first described gestational glucose intolerance, progressively lower thresholds have been proposed for the diagnosis and treatment of GDM. New data have demonstrated that the risk of adverse outcomes increases, even among women with sub-threshold results in fasting and post-load glucose screening [16]. Furthermore, it was recently reported that pregnant women with an isolated abnormal glucose value at 1-hOGTT carry a severe metabolic perturbation, characterized by markedly reduced beta-cell function; their metabolic phenotype resembles that of GDM, and may be associated with the same adverse outcomes as GDM [17]. The Hyperglycemia and Adverse Pregnancy Outcome (HAPO) study showed associations between increasing levels of fasting, 1-h, and 2-h plasma glucose obtained on oral glucose-tolerance testing and each of the adverse perinatal outcomes examined: birth weight above the 90th percentile and cordblood serum C-peptide level above the 90th percentile, premature delivery, shoulder dystocia or birth injury, intensive neonatal care, hyperbilirubinemia, and preeclampsia [18]. The effects of GDM on short-term breastfeeding outcomes have been clearly documented, unlike the effects of MIGT. Yet, women with a history of GDM face challenges with EBF in the critical period for setting up breastfeeding success. A recent systematic review of ten original papers from 1989 to 2013 on the effect of GDM on LII onset shows that all studies consistently identified that LII occurred later among gestationally diabetic mothers than in non-diabetic mothers [19]. LII is characterized by changes in breastmilk components—in particular, a decrease in breastmilk sodium. Conversely, the presence of GDM increases the risk of an elevated breastmilk sodium level on day three postpartum [20]. A case–control study revealed that women diagnosed with low milk supply were significantly more likely to have had GDM compared with women with other lactation outcomes [21]. Poorer sucking patterns have been found among newborn infants of mothers with GDM [22]. A cross-sectional analysis including 2038 women found that the adjusted odds of EBF at hospital discharge were lower among women with GDM compared to women without GDM [23].

Overt GDM is associated with significantly increased risks of adverse breastfeeding outcomes. Whereas, until recently, women with an abnormal 1-hOGTT (with normal 3h-OGTT) were regarded as a false positive result, emerging data suggest that current diagnostic criteria for GDM are too restrictive, and that lesser degrees of hyperglycemia also increase offspring risks [10]. Despite the associated adverse pregnancy outcomes, no international consensus exists that identifies a cut-off value for the definition of GDM. Current criteria of the International Association of Diabetes and Pregnancy Study Groups (IADPSG) are lower than the ACOG 2013 thresholds (fasting glucose 5.1 versus 5.8 mmol/L, or 1-h glucose 10.0 versus 10.6 mmol/L). It has been reported that applying the IADPSG criteria to the population would increase the rate of GDM from 7.3% to 10.3% [24]. A 2013 National Institutes of Health (NIH) panel states that there are clear benefits to the standardization of international diagnostic criteria. Nevertheless, the panel is concerned with the adoption of new criteria that would increase the prevalence of GDM (and the corresponding costs and interventions), without clear demonstration of improvements in health outcomes [25]. Therefore, new tools—such as Capula’s index—continue to be developed to improve the accuracy and cost-effectiveness of this screening [26].

A brief literature review shows that it is well established that insulin resistance is a strong predictor of short breastfeeding duration, but the underlying causal contributors remained unclear until very recently. Over the last years, insulin-sensitive gene expression has been shown to be upregulated during the lactation cycle, and insulin is now considered to play a direct role in lactation [27].

The established dogma has been that women are never physiologically unable to lactate, but rather that low milk supply is a mistaken belief on their part. Thus, most interventions have focused on improving breastfeeding education. However, emerging clinical research suggests an important association between suboptimal glucose tolerance and lactation difficulty. Our findings add to the evidence that a woman’s metabolic complications adversely affect her lactation outcomes. 1-hOGTT may be a useful clinical indicator to identify women at risk of early postpartum breastfeeding cessation. These results have clinical implications, underlining that breastfeeding support of women with abnormal glucose tolerance should be individualized. Given the current insulin resistance epidemic, caregivers face the challenge of meeting the growing need for clinical guidance of women with both metabolic disturbances and low milk supply. Further translational research is needed to successfully implement interventions that will enable more women to avoid undesired breastfeeding cessation.

These analyses have several limitations. First, this is a secondary analysis. Second, the nature of these data did not allow for evaluation of the contribution of interactions between conditions previously reported (e.g., gestational weight gain and maternal obesity, neonatal weight loss and DLII). Third, our significant findings may be inauthentic due to unmeasured confounding. A factor that influences a woman’s ability to breastfeed is the presence of personal or professional support. Other mother-centered factors include social barriers, work-related barriers, or a dislike of breastfeeding. We do not have data on all maternal factors that may affect milk production and composition. Finally, our assessment of metabolic dysregulation was done prenatally. We do not know to what extent maternal metabolic status in the early postpartum is affected.

Strengths of this study include the consistency with existing literature and the prospective design to collect breastfeeding duration and exclusivity data. The study does not rely on later recall of breastfeeding outcomes. Although the study populations and availability of pertinent confounders differed, the current study confirms that 1-hOGTT represents an important metabolic perturbation in pregnancy, characterized not only by increased risk for adverse pregnancy outcomes, but also by early breastfeeding dysfunction. It is becoming clear that long glucose challenge tests are not superior to short glucose challenge tests in the assessment of prenatal glucose tolerance. 

Finally, we clearly identified several additional independent risk factors for early breastfeeding cessation (e.g., neonatal weight loss, admission to the neonatal ward, and parity).

## 5. Conclusions

In summary, women who test positive for 1-hOGTT may be less able to sustain EBF at 100 days. These findings suggest that 1-hOGTT may already identify a high-risk population of mothers in need of interventions to increase breast milk production.

## Figures and Tables

**Table 1:** Patient characteristics.

Variable	NGT: 1-h Plasma Glucose < 7.8 mmol/L (*N* = 616)	MIGT: 10.6 mmol/L > 1-hOGTT Results ≥ 7.8 mmol/L (*N* = 152)	*p*
Gender:			0.72
Male	51%	53%	
Female	49%	47%	
Parity:			1
1	64%	65%	
>1	36%	35%	
Mother’s age (years)	33 (20–45)	33 (25–42)	0.064
Gestational weight gain	12 (1–39)	12 (4–27)	0.84
Weeks of gestation	40 (37–42)	40 (37–42)	0.79
Delivery type:			0.67
Eutocic	82%	18%	
Instrumental	80%	20%	
C-section	79%	21%	
Birth weight	3272 (1995–4800)	3395 (2050–4390)	0.018
Birth height	49.5 (33–54)	50 (45.5–53.5)	0.13
Birth head circumference	34.5 (31–37)	34.5 (31–37.5)	0.74
Percent of loss of birth weight to discharge	6 (−0.32–0.21)	7 (−7–13)	0.41

Abbreviations: 1-h post-glucose challenge (1-hOGTT); mild impairment of glucose tolerance (MIGT); normal glucose tolerance (NGT).

**Table 2:** Results for univariate analysis of exclusive breastfeeding (EBF) duration.

EBF Discontinuation	Before Day 100 (*N* = 384)	Equal or Later than Day 100 (*N* = 384)	*p*
MIGT	58%	42%	0.03 *
NGT	48%	52%	
Gender:			0.001 **
Male	214 (54%)	169 (43%)	
Female	179 (45%)	225 (57%)	
Parity:			<0.001 ***
1	271(72%)	214 (57%)	
>1	107 (28%)	159 (28%)	
Mother’s age (years)	33 (18–42)	33 (21–45)	0.92
Gestational weight gain	12 (1–30)	12 (4–39)	0.05
Weeks of gestation	40 (37–42)	40 (37–42)	0.27
Delivery type:			0.38
Eutocic	184 (50%)	207 (57%)	
Instrumental	61 (17%)	55 (15%)	
C-section	119 (33%)	110 (30%)	
Birth weight	3252 (1995–4390)	3330 (2310–4800)	0.02 *
Birth height	49.5 (33–54)	50 (45.5–54)	0.32
Birth head circumference	34.5 (31–37)	34.5 (31–37.5)	0.36
Percent of loss of birth weight to discharge	7 (−7–21)	6 (−32–20)	<0.001 ***

Abbreviations: mild impairment of glucose tolerance (MIGT); normal glucose tolerance (NGT). * *p* < 0.05; ** *p* < 0.01; *** *p* < 0.001.

**Table 3:** Multivariate analysis of factors independently associated with reduction in exclusive breastfeeding duration.

	AOR (95% CI)	*p*
Mildly impaired glucose tolerance	1.65 (1.11–2.45)	0.01
Early neonatal weight loss	1.73 (1.26–2.36)	0.001
Admission to neonatal ward	3.32 (1.04–10.60)	0.04
Parity	0.57 (0.41–0.79)	0.001

Abbreviations: AOR, adjusted odds ratio.

## Abbreviations

The following abbreviations are used in this manuscript:

1-hOGTT	1-h post-glucose challenge
3-hOGTT	3-h glucose tolerance test
ACOG	American College of Obstetricians and Gynecologists
DLII	delayed onset of lactogenesis stage II
EBF	exclusive breastfeeding
GDM	gestational diabetes
IADPSG	International Association of Diabetes and Pregnancy Study Groups
LII	lactogenesis stage II
MIGT	mild impairment of glucose tolerance
NGT	normal glucose tolerance
